# Dentistry 5.0: An Emerging Framework Integrating Bioengineering, AI, and Global Innovation Pathways for Equitable Oral Health

**DOI:** 10.7759/cureus.102000

**Published:** 2026-01-21

**Authors:** Andrea Mascolo, Giuseppe Bugelli, Oana Dinculescu, Matt J Kipper, Somayeh Baghersad

**Affiliations:** 1 Academics, European Institute for Medical Studies, Malta, MLT; 2 EIMS Research Hub, European Institute for Medical Studies (EIMS), St. Julian’s, MLT; 3 Biomedical Engineering, Colorado State University, Fort Collins, USA; 4 School of Biomedical Engineering, Colorado State University, Fort Collins, USA

**Keywords:** artificial intelligence in dentistry, biomedical engineering in dentistry, dentistry 5.0, digital health, global oral health, health equity, oral–systemic health, reverse evidence-based dentistry, translational dentistry

## Abstract

Dentistry is undergoing a profound transformation driven by advances in bioengineering that integrate artificial intelligence (AI), regenerative biology, digital manufacturing, salivary diagnostics, and extended reality (XR). Despite the rapid pace of innovation, global disparities in infrastructure, research capacity, and workforce distribution continue to limit the equitable adoption of these technologies. The emerging paradigm of Reverse Evidence-Based Dentistry (rEBD) provides a framework to link real-world clinical practice with scientific validation, strengthening translational pathways across diverse health systems. This narrative review analyzes the global intersection between dentistry and bioengineering, examining how innovations in AI-assisted diagnostics, biomaterials, regenerative platforms, wearable technologies, and XR-based education influence oral-systemic health, translational research, and the development of Dentistry 5.0. A secondary objective is to propose policy, educational, and research strategies that support equitable implementation across high-income countries (HICs) and low- and middle-income countries (LMICs), in alignment with rEBD principles. The review draws on international oral health data, biomedical engineering literature, global innovation studies, and academic collaborations, integrating evidence on regenerative dentistry, digital ecosystems, AI-driven diagnostics, frugal innovation for low-resource settings, and open-access scientific infrastructures. The findings indicate that bioengineering is reshaping dental practice through intelligent diagnostic systems, bioactive and regenerative materials, soft robotic devices, and digital manufacturing workflows, while AI- and XR-enhanced education platforms improve access to high-quality training, particularly in regions with limited infrastructure. Regional clinical hubs in LMICs facilitate context-specific validation of emerging technologies and support frugal innovation pathways, although persistent inequities remain in digital infrastructure, AI dataset diversity, regulatory readiness, and the integration of oral health into broader non-communicable disease strategies. Overall, Dentistry 5.0 reflects the convergence of biological, digital, and intelligent systems to deliver personalized, preventive, and globally connected oral healthcare, and its equitable implementation will require coordinated strategies grounded in the rEBD framework to ensure scientific rigor and global relevance.

## Introduction and background

Dentistry has evolved beyond traditional procedures to incorporate precision biomaterials, digital imaging technologies, artificial intelligence (AI), and stem cell-based regenerative platforms [1-4]. As oral diseases continue to affect over 3.5 billion people worldwide [5-7], dentistry increasingly intersects with bioengineering, a discipline that applies engineering principles to biological systems to develop innovative devices, materials, and diagnostic tools. For example, bioengineered implant surfaces and regenerative biomaterials have been shown to improve osseointegration, reduce inflammatory complications, and enhance long-term clinical outcomes, illustrating the direct translational impact of bioengineering on oral health outcomes, as consistently reported in contemporary reviews and consensus statements [4-7].

This convergence is redefining how oral health challenges are addressed globally, particularly in relation to prevention, therapeutics, diagnostics, and personalized care. This article expands on the lecture “Exploring the Intersection of Dentistry and Bioengineering: A Global Perspective,” delivered at Colorado State University, and examines how innovation, regulation, global collaboration, and digital transformation are reshaping dental technologies and their real-world implementation. By integrating insights from biomedical engineering, public health, digital dentistry, and translational science, we explore how bioengineering facilitates the movement of innovations from bench to chairside across diverse healthcare landscapes.

The central questions guiding this analysis focus on how bioengineering can enhance oral and systemic health outcomes [8-10], as well as on the regulatory, infrastructural, and cultural barriers that continue to restrict equitable access to dental innovation [11-14]. Particular attention is given to whether international and interdisciplinary collaboration can help bridge the innovation delivery gap between high-income countries (HICs) and low- and middle-income countries (LMICs) [15-20], and to how ethical, societal, and educational considerations shape the deployment of emerging technologies, including artificial intelligence, extended reality, and biosensing platforms [3,21-24].

These questions are not theoretical; oral diseases remain among the most prevalent non-communicable conditions worldwide, disproportionately affecting underserved populations and regions with limited access to trained providers and digital infrastructure [11-14].

To interpret these dynamics, this article adopts the conceptual lens of Reverse Evidence-Based Dentistry (rEBD), an emerging paradigm that begins with real-world clinical practice and technological innovation and then works backward to evaluate, align, and integrate the supporting scientific evidence [25]. By emphasizing feedback loops between clinical application, observational outcomes, and scientific validation, rEBD bridges the traditional gap between practice and evidence generation, offering a pragmatic framework particularly suited to digital, translational, and bioengineering-driven dentistry. Within this context, rEBD becomes a reflective and operational approach through which bioengineering, digital dentistry, and global health innovation can be critically analysed, implemented, and taught [25-27].

In this article, rEBD is used to align real-world technological innovations with the study objectives, guiding the analysis of translational pathways that connect clinical practice, evidence generation, and global implementation. To address these guiding questions, the following sections analyse the oral-systemic interface, emerging bioengineering innovations, global validation models, educational transformations, market dynamics, and ethical considerations. Each thematic section is organized into conceptual subsections to facilitate clarity and depth.

## Review

Materials and methods

This study is a narrative synthesis designed to integrate current evidence on bioengineering, digital dentistry, global innovation models, and the emerging framework of rEBD. The methodology followed standard approaches for narrative reviews, with an emphasis on conceptual integration, thematic clustering, and interdisciplinary interpretation rather than quantitative meta-analysis.

A structured literature search was conducted between January and December 2025 across multiple databases, including PubMed, Scopus, Web of Science, IEEE Xplore, World Health Organization (WHO) repositories, and leading biomedical engineering databases. Additional gray literature was retrieved from WHO Global Oral Health Reports, international innovation frameworks, and open-access repositories such as the Open Science Framework (OSF) and Zenodo. The search strategy employed combinations of keywords related to bioengineering, digital dentistry, regenerative dentistry, salivary diagnostics, artificial intelligence in dentistry, extended reality-based dental education, frugal innovation, clinical hubs, and Dentistry 5.0.

Sources published primarily within the previous 10 to 12 years were considered eligible, with earlier foundational studies included when necessary to contextualize specific technological domains such as stem cell biology, biomaterials, and early artificial intelligence systems. Eligible documents comprised narrative and systematic reviews, consensus reports, observational studies, translational research articles, global oral health policy documents, and innovation analyses relevant to dentistry and biomedical engineering. This time frame was selected to capture the period of most rapid technological acceleration in digital dentistry, bioengineering, and artificial intelligence-driven innovation while allowing the inclusion of seminal studies required to support conceptual continuity.

Publications without relevance to oral health, studies lacking methodological rigor, and non-peer-reviewed opinion pieces were excluded unless they provided essential conceptual insights, such as foundational frameworks on frugal innovation. Relevance and quality were assessed based on methodological transparency, consistency with the study objectives, clarity of technological or clinical application, and alignment with established scientific standards in dentistry and biomedical engineering, including consensus-based guidance, reproducibility of methods, and coherence with translational objectives.

Evidence was synthesized into five thematic domains encompassing global innovation ecosystems, oral-systemic health interfaces, bioengineering advances, digital education and equity, and future trajectories in Dentistry 5.0. Themes were identified through iterative reading, comparative analysis, and conceptual clustering, with priority given to cross-disciplinary integration and translational relevance.

The Reverse Evidence-Based Dentistry paradigm was applied as the interpretive framework guiding the synthesis. This approach begins with real-world technological and clinical innovations and works backward to evaluate, align, and integrate the supporting scientific evidence. Within this study, rEBD was used to connect practice-based observations, global disparities, and translational pathways across diverse health systems, providing a coherent structure for interpreting the reviewed evidence.

Results

Global Innovation and Collaboration Models

Global collaboration emerged as a central driver of advancement in dental bioengineering. Cross-continental academic partnerships linking institutions in North America, Europe, Asia, Africa, and Latin America enabled shared datasets, joint training programs, and multicenter validation of diagnostic and regenerative technologies. These alliances facilitated the translation of innovations such as salivary diagnostics, artificial intelligence-assisted imaging, and advanced biomaterials into diverse clinical environments.

Across multiple settings, collaborative initiatives supported the validation and deployment of diagnostic and regenerative technologies through multicenter pilot studies, shared educational programs, and implementation within regional clinical hubs. These initiatives were frequently associated with improved access to care, earlier diagnosis, and reduced logistical or operational costs, particularly in low-resource or decentralized healthcare environments. Collaborative networks also fostered the development of frugal innovation approaches that emphasized affordability, contextualized usability, and resource efficiency. Examples included offline-capable diagnostic platforms, mobile dental units, and low-cost three-dimensional printed prosthetic components specifically designed for low-resource settings. Collectively, these models reflected a shift from top-down technology transfer toward bottom-up co-creation grounded in local clinical realities.

Oral-Systemic Health Interface and Non-communicable Diseases (NCDs)

The findings confirmed strong bidirectional associations between periodontal inflammation and a range of systemic conditions, including diabetes, cardiovascular disease, adverse pregnancy outcomes, neurodegenerative disorders, and chronic kidney disease. Three major biological mechanisms underpinning these associations were identified, encompassing bacteremia that enables microbial dissemination, systemic inflammatory mediators that contribute to endothelial dysfunction, and molecular mimicry capable of amplifying autoimmune responses.

Salivary biosensors developed through bioengineering approaches exemplify the translational relevance of these mechanisms by enabling non-invasive detection of inflammatory and metabolic biomarkers associated with periodontal disease, supporting early identification of individuals at risk for systemic conditions such as diabetes or cardiovascular disease [5,6,21-23].

More broadly, bioengineering has contributed to the early detection of oral-systemic disease links through salivary diagnostics, biomarker biosensors, and wearable devices that allow real-time measurement of inflammatory, metabolic, and microbial markers. These platforms have demonstrated particular value in settings where conventional laboratory infrastructure is limited.

Emerging Bioengineering Innovations in Dentistry

Technological progress has reshaped prevention, diagnosis, and treatment across multiple domains of dental care. Materials science innovations, including high-strength ceramics, engineered titanium surfaces, and advanced polymers, such as polyetheretherketone, are now widely adopted in routine clinical practice due to improved biocompatibility, enhanced osseointegration, and reduced bacterial adhesion. In contrast, regenerative platforms encompassing stem cell-based therapies, growth factor-loaded hydrogels, and three-dimensional bioprinting remain largely in experimental or early translational phases, although they have expanded possibilities for pulp, periodontal, and alveolar bone regeneration.

Digital manufacturing workflows, such as chairside three-dimensional printing and artificial intelligence-assisted imaging diagnostics, have enhanced precision, reproducibility, and clinical efficiency. Wearable and bioelectronic devices have introduced new monitoring capabilities through intraoral biosensors, smart dentures, and mobile-linked diagnostic tools. Collectively, these innovations have accelerated the transition toward minimally invasive, data-driven, and personalized dentistry.

Clinical Hubs and Local Validation Models

Regional clinical research hubs, particularly in low- and middle-income countries, played a pivotal role in validating bioengineering innovations under real-world conditions. Unlike traditional centralized research models, decentralized hubs enabled the assessment of emerging technologies within community-based and primary-care dental settings. These hubs generated operational and usability data directly from underserved populations, facilitated early identification of infrastructural barriers, such as sterilization capacity and connectivity limitations, supported clinician training in emerging technologies, and enabled iterative feedback loops between practitioners and engineers.

Within this evolving ecosystem, emerging AI-driven robotic assistants for oral disease screening represent a further evolution of decentralized diagnostic models and may significantly enhance access to early diagnosis in mass-screening and low-resource settings, particularly in regions where dentist availability is limited.

In several documented implementations, decentralized validation models were associated with measurable operational benefits, including reductions in procedure time, improved workflow efficiency, and lower operational costs, such as shortened chairside diagnostic times and streamlined sterilization workflows, particularly in primary care and resource-limited dental settings [17-19]. These findings are consistent with reports from WHO-supported mobile oral health programs and decentralized diagnostic pilots conducted in low- and middle-income countries, which have demonstrated improved service delivery efficiency and reduced operational barriers in primary-care dental environments.

Comparative Models of Dental Care

The comparative dimensions summarized in Table 1 are derived from consolidated evidence reported by the World Health Organization, the Global Burden of Disease Oral Health Collaborators, and comparative analyses of dental care systems in high-income and low- and middle-income countries [10,11].

**Table 1:** Comparative overview of dental care models in high-income and low- and middle-income countries This table provides a conceptual comparison of dental care delivery models and system-level characteristics in high-income countries (HICs) and low- and middle-income countries (LMICs), highlighting differences in infrastructure, workforce availability, technology access, care organization, cost structure, and the role of bioengineering in supporting precision, scalability, and equity of oral healthcare. HICs, high-income countries; LMICs, low- and middle-income countries

Dimension	High-Income Countries (HICs)	Low- and Middle-Income Countries (LMICs)
Infrastructure	Advanced digital infrastructure	Limited/uneven infrastructure
Workforce	High dentist-to-population ratio	Workforce shortages
Technology access	Routine access to advanced diagnostics	Limited access; reliance on frugal innovation
Care delivery model	Centralized, specialist-driven	Decentralized, primary-care oriented
Cost structure	Public/insurance coverage	High out-of-pocket spending
Role of bioengineering	Optimization and precision	Access, scalability, affordability

Results showed substantial differences across global dental care systems, influencing technology adoption and equity. High-income countries typically displayed strong digital integration, higher dentist-to-population ratios, structured public coverage, and mature regulatory pathways for bioengineered solutions. Conversely, low- and middle-income countries often exhibited severe workforce shortages, fragmented access pathways, heavy reliance on out-of-pocket spending, and limited availability of advanced diagnostics and restorative technologies.

Digital Equity and Transformation in Dental Education

Digital education tools emerged as a critical factor influencing global access to high-quality dental training. Extended reality (XR) platforms improved psychomotor skill training and conceptual understanding, functioning even in low-bandwidth environments, and reducing dependency on physical simulation laboratories. Artificial intelligence-based educational systems provided real-time feedback, adaptive learning pathways, and multilingual support, expanding opportunities for learners in underserved settings. Open-access repositories, such as OSF, Zenodo, and PubMed Central, further enhanced global dissemination of training resources, supporting equitable participation in dental research and education.

Discussion

Digital Equity, AI, and Ethical Implications

The findings demonstrate that digital transformation in dentistry through XR simulation [28-30], artificial intelligence-driven learning systems [31-33], and open-access infrastructures [8] is increasingly central to global equity in oral health education. XR tools and virtual simulation platforms reduce the need for resource-intensive training environments and expand opportunities for students in low-bandwidth or geographically isolated regions [28-30]. Artificial intelligence tutors and adaptive learning systems further support inclusion by providing real-time feedback, personalized learning curves, and multilingual capabilities [31-33].

However, these innovations raise significant ethical considerations. Artificial intelligence applications in diagnostic imaging and periodontal assessment often rely on datasets that underrepresent populations from low- and middle-income countries, increasing the risk of algorithmic bias and unequal diagnostic performance [34-36]. Issues of data governance, privacy, and transparency also emerge as biosensors, salivary diagnostics, and wearable devices generate continuous biological data [6]. In low-resource settings, where digital infrastructure and data protection systems are limited, these concerns are amplified. Ethical implementation, therefore, requires attention to dataset diversity, explainable artificial intelligence, informed consent mechanisms, and the avoidance of “helicopter innovation,” where technologies are deployed without local co-design or capacity building [34-36].

Together, these considerations highlight the dual role of digital technologies as both enablers and potential amplifiers of inequity, underscoring the need for governance frameworks grounded in justice, transparency, and cultural sensitivity.

Market Dynamics and Global Growth Trends

The global dental market continues to expand, driven by demographic changes, improved economic conditions in emerging economies, and increasing integration of digital and regenerative technologies [37]. Key differences in technology adoption and equity gaps between high-income and low- and middle-income countries are summarized in Table 1, highlighting structural and infrastructural drivers of these disparities.

High-income countries lead the adoption of artificial intelligence diagnostics [31], digital imaging workflows [2], and regenerative materials [4-48], while low-resource regions emphasize frugal innovation models tailored to infrastructural constraints [38-41]. Asia-Pacific represents the fastest-growing market, with rapid advances in dental robotics and artificial intelligence-supported imaging [31], while North America drives investment in digital workflows and software integration [2]. European systems prioritize prevention, public coverage, and translational research [10]. Latin American and African regions show strong potential for growth but are constrained by workforce shortages, economic volatility, and limited integration of oral health into public health systems [11].

These dynamics influence where and how bioengineered innovations can achieve meaningful impact. Technologies designed for scalability, offline operation, or low maintenance show greater adoption in low-resource environments [38-41], while advanced materials and robotics remain concentrated in high-income systems [2,31]. Understanding these gradients is essential for designing innovation pathways that support global equity. In this context, frugal engineering translates market constraints into design drivers, enabling scalable, low-cost, and robust dental technologies that can be effectively deployed in low- and middle-income countries, where infrastructure, maintenance capacity, and workforce availability remain limited.

Dentistry 5.0: Future Trajectories

Dentistry is transitioning toward a model characterized by biological-digital convergence, predictive analytics, regenerative capabilities, and intelligent systems [3,4,48]. Artificial intelligence-powered decision support [31], extended reality for clinical planning [28-30], and multi-omics profiling are poised to deliver increasingly personalized and preventive care. Soft robotics, sensor-embedded implants, and self-monitoring devices reflect the emergence of minimally invasive, adaptive clinical ecosystems [6].

Dentistry 5.0 reframes oral healthcare as part of a broader network of systemic health monitoring and digital interoperability, aligning with global non-communicable disease frameworks [5-7,10]. This trajectory aligns naturally with rEBD, where practice-based innovations and real-world performance guide scientific inquiry, validation, and translation [25]. rEBD supports the development of practice-integrated laboratories that use digital tools, XR environments, and biosensing data to generate new forms of clinical evidence and accelerate translational research [25-27].

Policy Recommendations and rEBD Integration

The thematic synthesis indicates that achieving global equity in bioengineered dentistry requires multi-level action. Policymakers should integrate oral health into non-communicable disease agendas and universal health coverage strategies [10], prioritizing school-based prevention [11], mobile clinics, and low-cost diagnostics [38-41]. Academic institutions must embed interdisciplinary curricula linking dentistry with artificial intelligence, bioengineering, public health, and ethics [47,48], while expanding access to XR and artificial intelligence-based training in underserved regions [28-33].

These recommendations build directly on the results of this review, particularly the demonstrated role of decentralized clinical hubs, digital education platforms, and locally validated bioengineering models for improving access, feasibility, and sustainability across diverse healthcare settings. Industry stakeholders should adopt frugal engineering principles [38-41], co-create technologies with local clinicians, and support open-access knowledge ecosystems [8]. Global research consortia must invest in multi-ethnic datasets, transparent artificial intelligence pipelines, and South-South collaboration to strengthen scientific capacity in low- and middle-income countries [34-36].

Within this landscape, rEBD provides a unifying methodological framework that enhances scientific rigor while maintaining real-world relevance [25]. It ensures that emerging technologies are continuously aligned with global needs, ethical imperatives, and equitable implementation pathways.

Limitations of This Narrative Synthesis

This work is based on a narrative synthesis rather than a systematic review, which introduces several limitations. The search strategy, although structured, was not exhaustive, and publication bias may influence the availability of evidence from certain regions or disciplines. The descriptive nature of the included studies limits the ability to compare outcomes across heterogeneous methodologies. Additionally, the rapidly evolving landscape of artificial intelligence, regenerative dentistry, and digital health means that some developments may have emerged after the literature search [31-34,48].

Despite these limitations, the review provides a comprehensive interdisciplinary overview of current trends and identifies actionable pathways for global, equitable, and bioengineering-driven innovation in dentistry.

## Conclusions

Advances in bioengineering are transforming dentistry into a field increasingly defined by intelligent systems, regenerative solutions, and digitally supported clinical pathways. The convergence of artificial intelligence, biomaterials, salivary diagnostics, biosensing devices, and extended reality training is accelerating the evolution toward Dentistry 5.0, a model that emphasizes precision, prevention, and personalization. These developments offer significant opportunities to improve oral and systemic health outcomes while expanding access to high-quality care. However, the findings also underscore persistent global inequities in infrastructure, digital readiness, and research participation, particularly in low- and middle-income countries, highlighting the need for context-specific validation through regional clinical hubs, frugal engineering pathways, and collaborative networks that bridge academic, clinical, and industry stakeholders.

Within this context, Reverse Evidence-Based Dentistry (rEBD) emerges as a unifying methodological and educational framework capable of strengthening translational pathways. By grounding innovation in real-world clinical practice and iteratively aligning it with scientific evidence, rEBD enhances transparency, scientific rigor, and global applicability. A coordinated international effort integrating policy, education, research, and industry is therefore essential to ensure that the benefits of Dentistry 5.0 are implemented equitably and contribute to a more inclusive and sustainable global oral health future.
